# Neurophysiotherapy in Grade II Diffuse Astrocytoma: A Case Report

**DOI:** 10.7759/cureus.53082

**Published:** 2024-01-27

**Authors:** Simran F Sheikh, Aditi Akhuj, Raghumahanti Raghuveer, Akshaya Saklecha

**Affiliations:** 1 Department of Neuro-Physiotherapy, Ravi Nair Physiotherapy College, Datta Meghe Institute of Higher Education and Research, Wardha, IND

**Keywords:** physiotherapy, craniotomy, grade 2 glioma, tumour, diffuse astrocytoma

## Abstract

Diffuse astrocytoma is a slow, progressive, and invasive tumor that develops from astrocytes and there is no discernible boundary between tumor and brain cells. We present a case of a 48-year-old woman with diffuse astrocytoma who experienced sudden left-sided weakness, multiple convulsive episodes, and vomiting. The patient underwent surgery for a left occipital mini craniotomy with complete tumor removal through a titanium burr hole. Postoperatively, the patient complained of bilateral upper and lower extremities weakness, and decreased muscular tone was found; hence, she was referred to undergo neurophysiotherapy. A four-week rehabilitative protocol was started. Physiotherapy is critical in these patients for ensuring early and rapid recovery and treating the condition's clinical manifestations. The outcome measures employed were the tone grading scale, the Brunnstrom recovery stage, and the Functional Independence Measure (FIM). This case study concludes that physiotherapy rehabilitation for an operated case of grade 2 diffuse astrocytoma led to improved lower limb strength, normal tone, and improved functional independence, which helped the patient achieve better functional activities and a greater quality of life.

## Introduction

Gliomas are the most prevalent form of central nervous system (CNS) tumors; they originate from glial cells in the cerebrum, which are star-shaped [1]. The World Health Organization (WHO) has classified glioma into tumors of CNS and tumors of neuroepithelial tissue. Astrocytomas are the most typical variant of neuroepithelial tumors. They have been further classified as diffuse or isolated. Diffuse astrocytomas are innately invasive tumors that often emigrate along white matter networks deeply inside the normal brain. WHO classifies it as a grade II diffuse astrocytic neoplasm with a peak age of 30-39 years and a five-year survival rate ranging from 30 to 80% [2]. The regional impact of astrocytoma involves direct invasion and oxygen competition, which causes hypoxia of normal brain parenchyma, resulting in the release of free radicals, neurotransmitters, and inflammatory mediators, interfering with homeostasis and inducing clinical signs and symptoms [3]. Persistent headaches that exacerbate in the morning (a sign of increased intracranial pressure), vision distortion, speech difficulties, impaired cognition, and recent seizures are symptoms of diffuse astrocytoma [4]. When compared with a CT scan, MRI is a more sensitive imaging study for distinguishing low-grade tumors and soft tissue in general [5]. Currently, the standard treatment regimen includes surgery, radiotherapy, chemotherapy, and rehabilitative therapy [6]. Immediately following resection surgery for malignant brain tumors, patients could experience neurosurgical complications that could compromise their rehabilitation journey [7].

Physical therapy (PT) is a vital component of postoperative rehabilitation as it helps patients regain strength, mobility, and confidence. Neurophysiotherapy involves integrative techniques with a goal-oriented therapeutic regimen that includes neurodevelopmental treatment principles, passive stretching, weight-bearing exercises, and task-oriented strategies. These treatments improve gross motor functions, and static and dynamic balance, and help with spasticity reduction, muscle tone enhancement, and independent walking. After the operation, the rehabilitation should involve a tailored plan aimed at restoring muscle strength and joint function, enhancing mobility, and providing the patient with the confidence to live an active life [8]. The rehabilitative intervention has an enormous effect on the patient's motor function, perception, psychology, and quality of life [9]. The key objective of physical therapy is to enhance the patient's motor skills, muscle tone, mobility, perception of sensation, and self-reliance.

## Case presentation

Patient information

A 48-year-old female presented with complaints of multiple episodes of seizures, unrelenting headaches, and vomiting without any history of trauma or loss of consciousness. Her guardian reported a previous incidence linked to such complaints, and a previous MRI scan performed three years ago showed that the patient had an insular glioma, for which frontal craniotomy with glioma excision had been carried out. After the medical examination, the patient was referred for further evaluation, comprising an MRI and CT scan, which confirmed diffuse astrocytoma grade 2 in the frontoparietal lobe. She eventually underwent surgery for left frontoparietal craniotomy with tumor resection. After the procedure, the patient was moved to the neuro-intensive care unit. Postoperatively, she was unable to move her upper and lower limbs due to muscle weakness. Physiotherapy rehabilitation was implemented with a peculiar maneuver for the patient.

Clinical findings

Before the examination, the patient was briefed about the process, and her consent was obtained. On the day of the assessment, the patient was placed in a supine position with her head elevated to 30 degrees. She was conscious, oriented, and responsive to vocal commands, and was of a mesomorphic build. The Glasgow Coma Scale score was 15/15 on neurological examination, and a higher mental function test was conducted [the mini-mental state examination (MMSE) result was 26/30]. Cranial nerves V, VII, and VIII had been impaired. The sensory examination showed bilaterally intact sensations. During the motor examination, the tone assessment indicated the presence of grade 1+ tone (tone grading scale) in the left upper and lower limbs, as shown in Table 1. The assessment of the superficial and deep reflexes is demonstrated in Table 2. The muscle strength in both the left upper and lower limbs was 2/5. Outcome measures were gauged on day one of the assessment as the Brunnstrom recovery stage score was 2, and the Functional Independence Measure (FIM) observed was 69.

**Table 1:** Pre-intervention muscle tone (TGS) 1+: decreased tone; 2+: normal tone; 3+: increased tone TGS: tone grading scale

Muscles	Right	Left
Shoulder		
Flexors	2+	1+
Extensors	2+	1+
Elbow		
Flexors	2+	1+
Extensors	2+	1+
Hip		
Flexors	2+	1+
Extensors	2+	1+
Knee		
Flexors	2+	1+
Extensors	2+	1+
Ankle		
Dorsiflexors	2+	1+
Plantarflexors	2+	1+

**Table 2:** Pre-intervention deep tendon reflexes +: diminished reflex; ++: normal reflex; +++: exaggerated reflex

	Right	Left
Bicep jerk	++	+
Tricep jerk	++	+
Knee jerk	++	+
Ankle jerk	++	+

Diagnostic assessment

**Figure 1:**
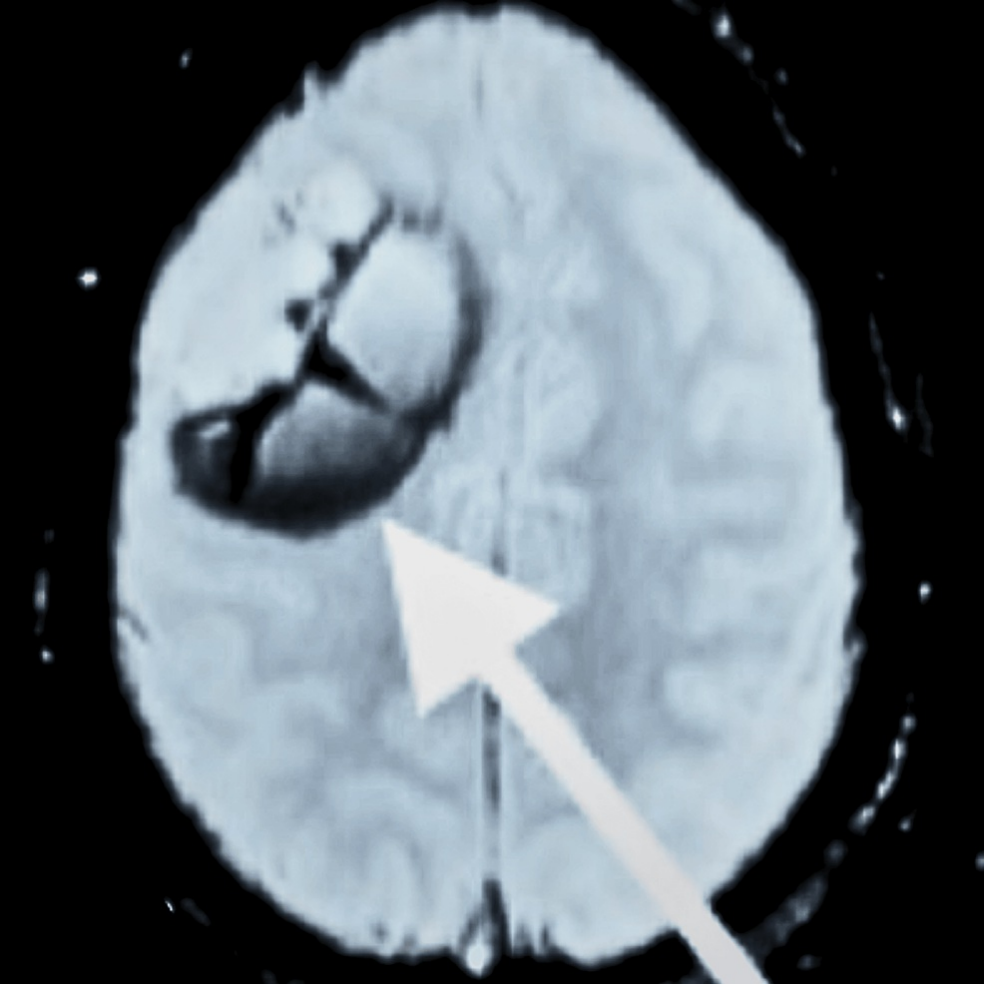
MRI of the brain The white arrow shows a well-defined, space-occupying lesion MRI: magnetic resonance imaging

The patient's brain MRI was carried out via T1, T2, fluid-attenuated inversion recovery (FLAIR), and diffusion-weighted imaging (DWI) with contrast-enhanced gadolinium contrast medium (Ce-Gd study), which showed diffuse astrocytoma grade 2 in the right frontoparietal lobe. The MRI revealed a well-defined, space-occupying lesion in the right frontoparietal lobe extending in the right Sylvian fissure, measuring approximately 8.5 × 6.6 × 5.5 cm in anteroposterior, transverse, and craniocaudal dimensions (Figure 1).

Physiotherapy intervention

**Figure 2:**
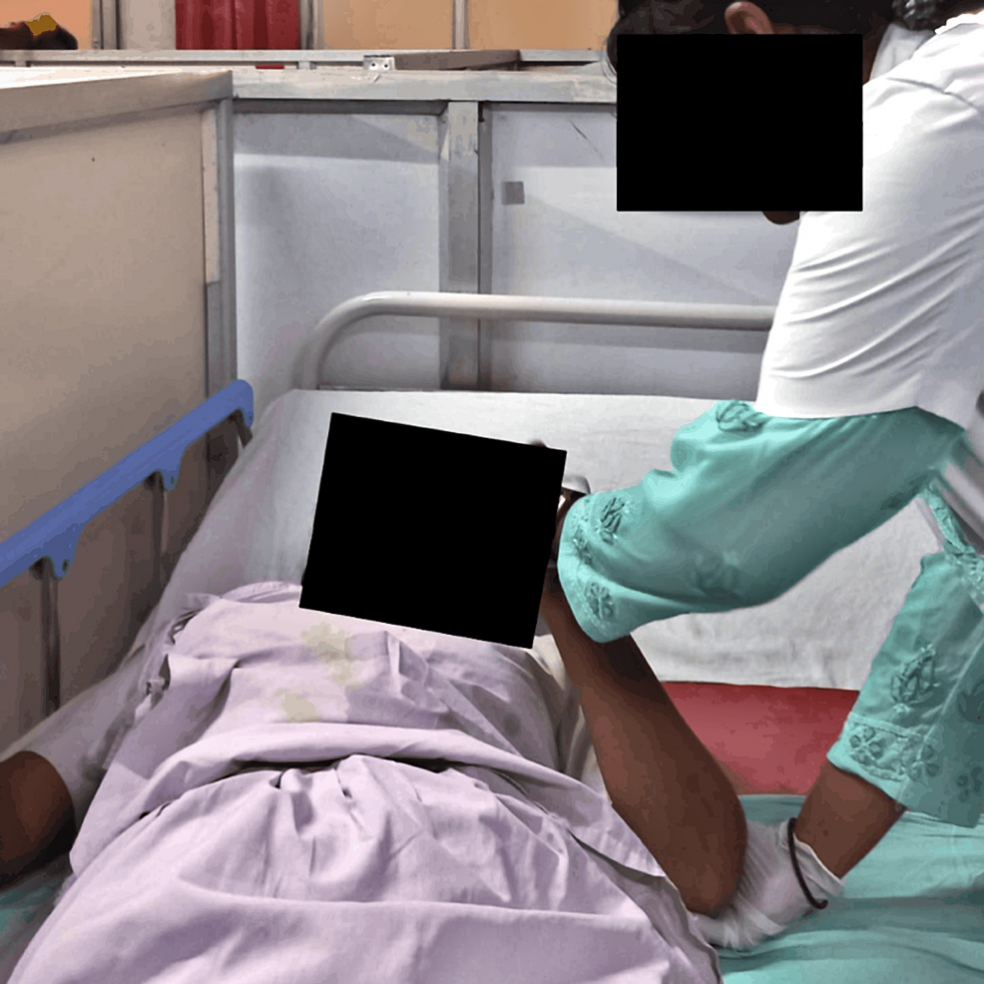
Therapist performing PNF in the upper limb PNF: proprioceptive neuromuscular facilitation

**Figure 3:**
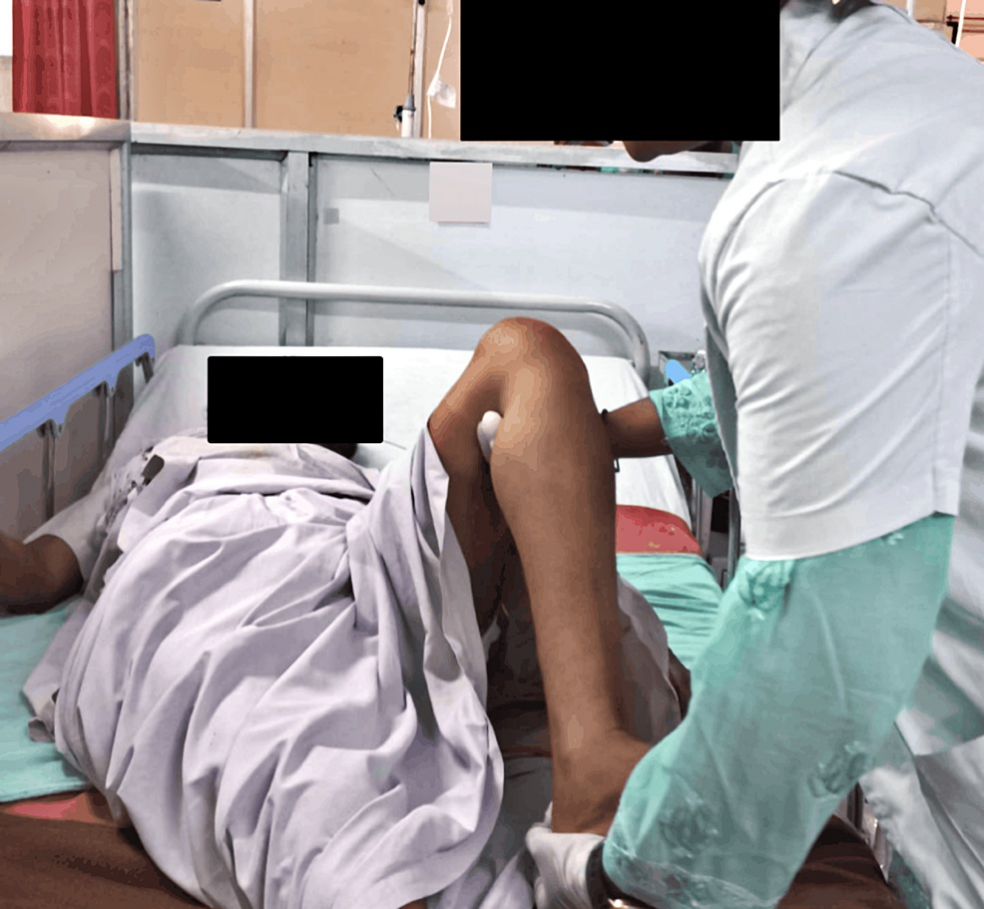
Therapist performing PNF in the lower limb PNF: proprioceptive neuromuscular facilitation

Initially, the patient was managed through surgical procedures; her craniotomy was done with glioma evacuation, and currently, she is on medications. Postoperatively, she suffered from muscle weakness along with a decrease in muscle tone. The key objective of rehabilitation was to help her regain her functioning skills and self-reliance. Table 3 summarizes the physiotherapeutic protocol, and Figures 2-3 illustrate the rehabilitation measures administered to the patient.

**Table 3:** Physiotherapy protocol* *[10] PNF: proprioceptive neuromuscular facilitation; UL: upper extremity; LL: lower extremity; ROM: range of motion

Goals	Therapeutic intervention	Treatment protocol
To promote bed mobility and functional activities	Supine-to-sit transition training with rolling facilitation	The initiation of the rolling process was carried out by rolling from supine to side-lying and crossing both ankles. Transitioning training from supine to sitting, prone on elbows to long sitting, using elbow walking, supine on the elbows
To restore muscle tone	PNF to UE and LE	Rhythmic initiation technique: 10 repetitions with 2 sets. Progression of the PNF, combinations of isotonics to slow reversal: 10 repetitions with 2 sets
To prevent contractures	Stretching and ROM exercises for the right upper and lower limb	Progressive and uniform stretch for 30-second hold with 3 repetitions and 1 set. ROM exercises (10 repetitions for 2 times a day)
To enhance functional skills and prepare for sit-to-stand transition	Bedside sitting	Bed mobility, supine to sitting and then to side lying. The patient sits with her feet supported, her hips and knees are in 90-degree flexion, her torso is in a neutral position, and her elbows are locked
To strengthen lower limb muscles	Sit to stand	Sit to stand with a 30-second hold, then progress to a 60-second hold: 5 repetitions for 2 times a day

Follow-up and outcomes measures

The rehabilitation measures improved the patient’s muscle tone and significantly helped in enhancing the patient's functional abilities and recovery as per the Brunnstrom stage of recovery. After the end of therapy, the patient gained the strength to carry out elementary functional tasks such as sitting by the bed without assistance and standing with minimal support. Post-rehabilitative muscle tone and reflex examination are summarized in Tables 4-5. The muscle strength in both the left upper and lower limbs was 4/5.

**Table 4:** Pre- and post-intervention muscle tone 1+: decreased tone; 2+: normal tone; 3+: increased tone

Extremity	Pre-intervention		Post-intervention	
	Right	Left	Right	Left
Upper limb	2+	1+	2+	2+
Lower limb	2+	1+	2+	2+

**Table 5:** Pre- and post-intervention deep reflex +: diminished reflex; ++: normal reflex; +++: exaggerated reflex

Reflex	Pre-intervention		Post-intervention	
	Right	Left	Right	Left
Bicep jerk	++	+	++	++
Tricep jerk	++	+	++	++
Knee jerk	++	+	++	++
Ankle jerk	++	+	++	++

The findings related to outcome measures are shown in Table 6. 

**Table 6:** Outcome measures

Outcome measures	Pre-intervention	Post-intervention
Tone grading scale	1+	Normal tone
Brunnstrom recovery stage	2	5
Functional Independence Measure	69/126	110/126

## Discussion

In contrast with almost all typical brain cancers, diffuse gliomas are distinguished by a wide-ranging, diffuse infiltration of tumor cells inside the neuropil, which is the intricate web of interconnected neuronal and glial cell processes. The invasive diffuse gliomas are difficult to visualize radiologically [11]. The cerebrum is the primary location of a majority of diffuse astrocytomas, but these tumors can occur in any part of the CNS, including the basal ganglia, brainstem, cerebellum, and spinal cord [12]. Histopathology categorizes grade II astrocytomas as uniform masses with varying borders caused by a suspected p53 gene mutation (causing it to be passed down through generations) and loss of chromosome 17 heterozygosity. The primary imaging technique to recognize and assess the brain tumor is MRI.

The initial and most commonly employed cure for individuals with brain tumors is a surgical procedure [13]. The complete resection of the tumor has been associated with a better prognosis, given that brain tumor boundaries are difficult to identify and surgery might impact brain function [14]. When dealing with grade II gliomas, early resection should be more desirable over cautious waiting. In most cases, the surgical removal of these kinds of tumors is not possible [15]. Additionally, neurological impairment occurs in 30% of patients postoperatively due to the tumor's close proximity to the brain stem and enlarged and soft cranial nerves (edema) during tumor removal [16]. The resection should be as extensive as achievable. Admission to the ICU, or critical care unit, following brain tumor surgery is common. In neurosurgical patients, no analgesic regimen has been proven to be effective for avoiding complications such as weakness and decreased muscle tone, due to which the patient is unable to move upper or lower limbs.

Research has shown that problems during the first 24 hours after brain tumor surgery are common, and patients often suffer from muscular weakness and motor impairment [17]. Hemiplegia is one of the most frequent neurological complications seen in patients after tumor resection, impacting functional independence and, consequently, an individual's quality of life [18]. The Australian Cancer Network (ACN) recommends physiotherapy for patients with persisting motor impairments (strength, balance, and coordination), along with personal care and self-reliance measures [19]. Therapeutic exercise can help with problems related to weakness, tense muscles, muscle tone, and stiff joints. Physical therapy-assisted exercise has been shown to strengthen cancer patients' quality of life in general. Physiotherapy has been empirically shown to boost muscular strength and endurance. Complete rehabilitation programs for motor, balance, cognition, and activities of daily living (ADL) function can lead to significant improvements in functioning in patients who have gone through a craniotomy procedure. Resistance training is recommended to enhance the strength of the muscles, while endurance exercises are advised to compensate for poor coordination. Patients who adopted the PNF technique during their course of rehabilitation therapy observed changes in their gait and daily activities. In general, the faster a person regains strength, the faster he or she recovers. The prevention of complications and optimizing the patient’s functional abilities are the two main objectives of postoperative rehabilitation [20].

The most important objective of early rehabilitation is to facilitate the patient’s immediate recovery and eliminate secondary complications like decreased muscular tone and muscle weakness. In this case report, we mainly aimed to highlight the significance of physiotherapy for patients who experience adverse effects after glioma removal and craniotomy.

## Conclusions

Neurophysiotherapy played a vital role in the postoperative rehabilitation of craniotomy in our patient who experienced secondary complications such as muscular weakness and decreased muscle tone. Through neurophysiotherapy interventions, significant improvements were achieved in strengthening muscles and regaining lost tone. It offers several benefits, including improved motor function, faster recovery, reduced complications, enhanced quality of life, and increased level of independence. The tailored rehabilitation programs provided by physiotherapists can help patients regain their strength, mobility, and overall well-being, making it an integral part of the comprehensive care for neurosurgical patients.

## References

[1] Kapoor M, Gupta V (2023). Astrocytoma. https://www.ncbi.nlm.nih.gov/books/NBK559042/.

[2] Pellerino A, Caccese M, Padovan M, Cerretti G, Lombardi G (2022). Epidemiology, risk factors, and prognostic factors of gliomas. Clin Transl Imaging.

[3] (2023). Diffuse astrocytoma (grade II). https://braintumorcenter.ucsf.edu/condition/diffuse-astrocytoma-grade-ii.

[4] (2023). Astrocytoma tumors. https://www.aans.org/en/Patients/Neurosurgical-Conditions-and-Treatments/Astrocytoma-Tumors.

[5] Aiman W, Gasalberti DP, Rayi A (2023). Low Grade Glioma. https://www.ncbi.nlm.nih.gov/books/NBK560668/.

[6] Kesari S (2011). Understanding glioblastoma tumor biology: the potential to improve current diagnosis and treatments. Semin Oncol.

[7] De la Garza-Ramos R, Kerezoudis P, Tamargo RJ, Brem H, Huang J, Bydon M (2016). Surgical complications following malignant brain tumor surgery: an analysis of 2002-2011 data. Clin Neurol Neurosurg.

[8] (2023). What is post surgery rehabilitation and why is it necessary?. https://www.miraclerehabclinic.com/blog/post-surgery-rehabilitation.

[9] Zhao K, Yu C, Gan Z, Huang M, Wu T, Zhao N (2020). Rehabilitation therapy for patients with glioma: a PRISMA-compliant systematic review and meta-analysis. Medicine (Baltimore).

[10] Kaple N, Harjpal P, Samal SS (2022). Neuro-physiotherapy regimen to enhance the functional performance of a hemiplegic patient following brain tumor resection: a case report. Cureus.

[11] Claes A, Idema AJ, Wesseling P (2007). Diffuse glioma growth: a guerilla war. Acta Neuropathol.

[12] Mclendon RE (2023). Pathology of diffuse astrocytomas definition and overview. Medscape.

[13] (2023). Brain tumor surgery. https://www.hopkinsmedicine.org/health/conditions-and-diseases/brain-tumor/brain-tumor-surgery#:~:text=Endoscopic%20Brain%20Tumor%20Surgery%20(Neuroendoscopy,called%20an%20endonasal%20endoscopic%20surgery.

[14] Chaulagain D, Smolanka V, Smolanka A (2024). Diagnosis and management of astrocytoma: a litreature view. Inter Neuro J.

[15] Louis DN, Ohgaki H, Wiestler OD (2007). The 2007 WHO classification of tumours of the central nervous system. Acta Neuropathol.

[16] Soliman S, Ghaly M (2021). Ischemic stroke after tumor resection in a patient with glioblastoma multiforme. Cureus.

[17] Picca A, Berzero G, Sanson M (2018). Current therapeutic approaches to diffuse grade II and III gliomas. Ther Adv Neurol Disord.

[18] Lee EJ, Lee SK, Agid R, Bae JM, Keller A, Terbrugge K (2008). Preoperative grading of presumptive low-grade astrocytomas on MR imaging: diagnostic value of minimum apparent diffusion coefficient. AJNR Am J Neuroradiol.

[19] Ching W, Luhmann M (2011). Neuro-oncological physical therapy for the older person. Top Geriatr Rehabil.

[20] Kos N, Kos B, Benedicic M (2016). Early medical rehabilitation after neurosurgical treatment of malignant brain tumours in Slovenia. Radiol Oncol.

